# Retrospective analysis of immediate in-brace correction of scoliosis attainable in patients with AIS: a SOSORT initiative

**DOI:** 10.1186/1748-7161-8-S1-O49

**Published:** 2013-06-03

**Authors:** P Knott, F Techy, T Cotter, L Jansen, P Kove, J Loving, K Poletis, S Mardjetlko

**Affiliations:** 1 Rosalind Franklin University, Chicago, IL, USA; 2 Illinois Bone and Joint Institute, Chicago, IL, USA; 3 3T Imaging

## Background

The effectiveness of bracing in AIS has been debated for years, and there are few studies that can provide a clear picture of how a brace influences curve magnitude, rate of progression, or reduction in surgery. A 2007 review article found no clear advantage to bracing, over observation, in reducing the need for surgery [1]. However, the article highlighted the lack of uniformity in the studies it reviewed, and the high variability in the outcomes that it pooled. A 2010 Cochrane Systematic Review also found that there was only low-quality evidence in favor of using braces [2]. One must first be able to distinguish effective from ineffective bracing, as there is no reason to evaluate the outcome of ineffective braces. A standard must be set as to the amount of immediate curve correction that a brace should deliver, before any brace treatment is labeled as effective or ineffective. Other authors have outlined the appropriate criteria for evaluating brace effectiveness, but have not included immediate in-brace correction of the curve in their list of outcome measures [3]. This study was developed as a SOSORT board initiative, and will attempt to develop that standard.

## Aim

The aim of this study is to evaluate the radiographs of patients who have just had a brace applied, and to determine the amount of correction that is routinely achieved.

## Methods

For this study, a group of European physicians, skilled at scoliosis bracing, were recruited to submit sequential pre- and post-bracing radiographs of their patients for a specified period of time. American physicians, experienced in scoliosis evaluation, measured the Cobb angles in the two sets of radiographs. The percent correction was calculated using these two measurements.

## Results

The results were stratified according to age, gender, initial curve magnitude, and brace type. Average curve corrections were calculated for each group.

## Conclusions

Although there is variation among the subgroups evaluated, an effective brace should be able to achieve 50% correction of the curve magnitude, immediately after application. Research that includes patients whose curves has significantly less than 50% correction in-brace are not studies of “effective” bracing.
